# The IntelliCage System: A Review of Its Utility as a Novel Behavioral Platform for a Rodent Model of Substance Use Disorder

**DOI:** 10.3389/fnbeh.2021.683780

**Published:** 2021-06-04

**Authors:** Ismail Nurul Iman, Nurul Aiman Mohd Yusof, Ummi Nasrah Talib, Nur Aimi Zawami Ahmad, Anwar Norazit, Jaya Kumar, Muhammad Zulfadli Mehat, Nanthini Jayabalan, Sangu Muthuraju, Marzena Stefaniuk, Leszek Kaczmarek, Mustapha Muzaimi

**Affiliations:** ^1^Department of Neurosciences, School of Medical Sciences, Universiti Sains Malaysia, Kubang Kerian, Malaysia; ^2^Department of Anatomy, School of Medical Sciences, Universiti Sains Malaysia, Kubang Kerian, Malaysia; ^3^Department of Biomedical Sciences, Faculty of Medicine, University of Malaya, Kuala Lumpur, Malaysia; ^4^Department of Physiology, Faculty of Medicine, Universiti Kebangsaan Malaysia, Kuala Lumpur, Malaysia; ^5^Department of Human Anatomy, Faculty of Medicine and Health Sciences, Universiti Putra Malaysia, Serdang, Malaysia; ^6^Translational Neuroscience Lab, UQ Centre for Clinical Research, The University of Queensland, Brisbane, QLD, Australia; ^7^Department of Pharmacological and Pharmaceutical Sciences, College of Pharmacy, University of Houston, Houston, TX, United States; ^8^BRAINCITY, Nencki Institute of Experimental Biology, Polish Academy of Sciences, Warsaw, Poland

**Keywords:** IntelliCage system, substance use disorder (SUD), addiction, rodent model, behavior, home cage

## Abstract

The use of animal models for substance use disorder (SUD) has made an important contribution in the investigation of the behavioral and molecular mechanisms underlying substance abuse and addiction. Here, we review a novel and comprehensive behavioral platform to characterize addiction-like traits in rodents using a fully automated learning system, the IntelliCage. This system simultaneously captures the basic behavioral navigation, reward preference, and aversion, as well as the multi-dimensional complex behaviors and cognitive functions of group-housed rodents. It can reliably capture and track locomotor and cognitive pattern alterations associated with the development of substance addiction. Thus, the IntelliCage learning system offers a potentially efficient, flexible, and sensitive tool for the high-throughput screening of the rodent SUD model.

## Introduction

Understanding the neural mechanisms of complex human behaviors and the behavioral anomalies accompanying neurobiological disorders, including substance use disorder (SUD; or substance addiction), represents one of the most formidable challenges in behavioral and cognitive neuroscience research at present (Lynch et al., 2010; Gulinello et al., 2019; Kuhn et al., 2019). The past decade has seen a resurgence of studies using laboratory rodents, coupled with an impressive array of genetic modifications, providing unprecedented opportunities to generate suitable rodent models to research human pathologies. In contrast, behavioral assays, and their application to large numbers of animals, trailed behind in terms of throughput if compared with genetic advances for rodent models. Notwithstanding, there is a growing need for reliable and robust high-throughput behavioral assessment platforms to elucidate the cognitive and behavioral performances in both wild-type and transgenic rodent strains.

To address this issue, we focus on the IntelliCage system, a home-cage-based rodent behavioral assessment platform, and specifically, its utility to investigate the neurobehavioral underpinnings of SUD in rodent models. This fully automated live-in environment approach helps eliminate the confounding effects and considerable stress from environmental and experimental variables that may obscure the behavioral measures. Owed to its low-dependency on human interference, the IntelliCage system also enables investigators to monitor the multi-dimensional processes in group-housed mice regulated over longer time scales in a straightforward, time-, and cost-effective manner (Lipp, 2005; Lipp et al., 2005; Spruijt and DeVisser, 2006; Wolfer et al., 2012; Kiryk et al., 2020). Such an approach may not only improve throughput, but also provide new insights into the regulation of rodent behaviors that is not as practical with conventional behavioral assays.

In this narrative review, we provide an overview on several commonly employed animal models of SUD, describe the IntelliCage system apparatus and its application in modeling human neurological disorders, and provide in-depth reviews of the related SUD studies utilizing the IntelliCage in the assessment of multi-symptomatic animal physiology, behaviors, and cognitive functions.

## Common Rodent Models of Substance Use Disorder

Substance use disorder is a chronically relapsing disorder characterized by compulsive and uncontrollable substance-seeking and use, which persists even in the face of negative consequences (Koob and Volkow, 2010, 2016; Uhl et al., 2019). Animal models of SUD are recognized as indispensable tools in defining our current knowledge of the neurobiology and pathophysiology of addiction, and the neuropharmacological aspects of substances of abuse (Koob, 2014; Venniro et al., 2016; Wingo et al., 2016; Müller, 2018; Kuhn et al., 2019). Although animal models may not fully emulate and reproduce the complex human experience, they nevertheless provide means for the researchers to conduct addiction research under highly controlled conditions that may not be possible or ethical to replicate in humans. Earlier animal models of SUD emphasized on the use of operant paradigms in non-human primates and the mechanisms of acute reward. However, recently, these paradigms have been extrapolated and utilized in small rodents (namely, laboratory mouse and rat). Current research has also shifted to include consequent neuroadaptations in long-term or chronic substance abuse paradigms. The use of rodent models, together with the recent advancement, has provided significant new knowledge and understanding in the neurobiology of SUD.

### Behavioral Sensitization

The behavioral sensitization model (i.e., experimenter-administered drug exposure) has been extensively used to assess drug-induced locomotor changes, and to identify key reward-related neurobiological substrates and the underlying neuroplasticity (Steketee and Kalivas, 2011; Kuhn et al., 2019). The model involves a progressive increase in the motor stimulatory effects that occur with a repeated, intermittent exposure to a specific drug. Depending on the experimenter’s timeline, sensitization can be rapidly induced to study the short-term drug-induced changes and/or long-term effects of chronic drug exposure. The development of behavioral sensitization has been hypothesized to represent a transition from drug “liking” to “wanting” that underlies compulsive substance use as reported to occur in response to morphine (Cheaha et al., 2017), amphetamine (Ridzwan et al., 2017), alcohol (Mitra and Nagaraja, 2020), nicotine, cocaine, and cannabinoids (Steketee and Kalivas, 2011; Venniro et al., 2016; Iman et al., 2017; Müller, 2018; Kuhn et al., 2019).

### Drug Self-Administration Paradigm

Current animal models of SUD emphasize on the addictive drugs actions as positive reinforcing stimuli, much like food, water, and other “natural” reinforcers. Laboratory animals can voluntarily self-administer these addictive substances leading to intoxication, which mimics the drug-taking behaviors seen in human addicts (Panlilio and Goldberg, 2007; Kuhn et al., 2019). In a commonly used paradigm, the animals (typically a mouse, rat, or monkey) are trained in an operant chamber to obtain a drug reward for short daily sessions (1–3 h), and even up to several months in a more complex chronic drug training. Drug delivery is made dependent on the performance of either a fixed or progressive ratio operant response; typically, lever press or nose-poke is used in rodents. Compared to the other models of SUD, these procedures provide the most likely representations with addictive behavior that occurs in the natural environment, as evidenced by the short-, intermittent, and/or long-access to emulate drug-taking and drug-seeking experimental designs. Hence, this self-administration paradigm has a high degree of face validity and is considered to be the gold standard in examining the reinforcing properties of addictive substances in rodents (Panlilio and Goldberg, 2007; Koob, 2014; Kuhn et al., 2019). Furthermore, this close correspondence allows the details of the procedure to be modified in a variety of ways to model specific aspects of addiction. The behavior observed is also highly sensitive to the manipulations of specific environmental and pharmacological variables. Thus, this makes the self-administration paradigm a suitable test for a better understanding of the factors to model drug seeking behavior leading to addiction, and they can also provide a means of testing potential therapeutic agents with anti-addictive properties or even evaluate the abuse potential of novel psychotropic candidates (Panlilio and Goldberg, 2007; Lynch et al., 2010; Spanagel, 2017).

### Conditioned Place Preference and Aversion Paradigm

Conditioned Place Preference (CPP) paradigm is a behavioral model commonly used to study the rewarding and/or aversive effects of natural and pharmacological stimuli, a learned behavior shown in many vertebrates, including humans (Huston et al., 2013). Although various designs and apparatuses are used to model CPP, the fundamental characteristic of this task involves the classical conditioning procedure where a particular environmental setting is associated with drug exposure, followed by the association of a different environment with the absence of the drug (or drug vehicle). After several environmental pairings, the drug-free animal is allowed to freely access both ends of the CPP paradigm, where the time spent in each environment will be measured. Theoretically, when addicted, the animals will exhibit a CPP for the environment paired with the drug reward that functions as a positive-reinforcer (i.e., spend more time in drug-paired vs non-drug environment) and avoid those that induce aversive states [i.e., conditioned place aversion (CPA)], frequently cued by a foot-shock punishment. This procedure permits the assessment of the conditioning of drug reinforcement, and provides information regarding the positive and negative reinforcing effects of drugs besides being relatively easy, quick, economical, and reproducible (Aris et al., 2012; Huston et al., 2013; Koob, 2014). Commonly abused substances such as morphine (Gibula-Tarlowska et al., 2019), cannabis (Clasen et al., 2017), amphetamines (Bardgett et al., 2020), cocaine (Carmack et al., 2013), nicotine (Muldoon et al., 2020), ethanol (Campos-Jurado et al., 2020), and 3,4-methylenedioxymethamphetamine (MDMA; Rodríguez-Arias et al., 2013) have been shown to readily establish a CPP and CPA paradigm in rodents. This paradigm is also considered a common and useful screening tool to assess the abuse liability of novel drugs due to its relative ease, economic, and reproducible set-ups (Huston et al., 2013).

## The Intellicage System

**FIGURE 1:**
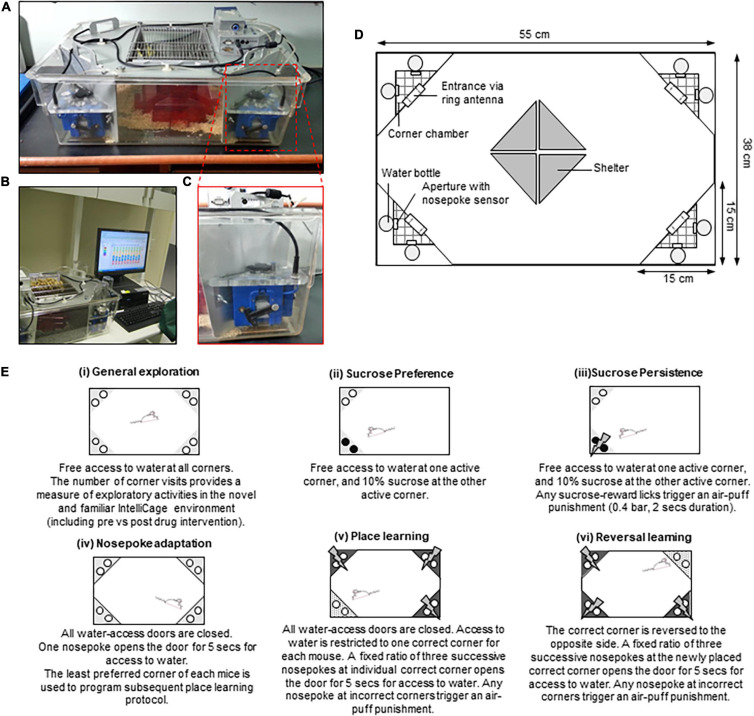
An overview of the IntelliCage system. **(A)** The IntelliCage apparatus. **(B)** The IntelliCage apparatus is connected to a computer-based software used to design various behavioral protocols, as well as to measure and analyze mice behavioral patterns. **(C)** The motorized doors at each IntelliCage corner chamber which control access to water bottle nipples. **(D)** Schematic illustration of the IntelliCage. **(E)** Summary of IntelliCage parameters modified from Iman et al. (2017).

The IntelliCage system (Figure 1) is a social-group environment developed by Hans-Peter Lipp and colleagues of the University of Zurich, Switzerland primarily for the use of Neural Plasticity & Repair, National Centre for Competence in Research (NCCR) research groups (Lipp, 2005; Lipp et al., 2005; Kiryk et al., 2020). IntelliCage is the first fully automated cage system designed for the assessment of spontaneous activity, spatial learning, memory, and cognitive abilities of rodents living in social groups. It allows the individual recording of the long-term and multi-dimensional behavioral patterns of up to 16 animals simultaneously. Various experimental paradigms and protocols can be freely programmed and executed with this system, thus, allowing maximum flexibility in the experimental design. Data are recorded while the animals are housed in the IntelliCage system, which provides considerably more information for analysis compared to any conventional method. The IntelliCage system was designed to circumvent practical issues often encountered with the standard behavioral test paradigms (as summarized in Table 1). The automated generation and collection of data by standardized procedures allow for high data comparability and reproducibility between labs thereafter, permitting a reduced number of animal replications needed to obtain reliable findings. This system also minimizes the need for human or experimenter’s handling, thus, reducing external artifacts that interfere with the animals’ activities throughout the desired period of monitoring (Lipp, 2005; Lipp et al., 2005; Kiryk et al., 2020).

**TABLE 1:** Summary of refinement by the IntelliCage system compared to standard behavioral paradigms.

Standard behavioral Paradigms	Refinement by the IntelliCage system
Radial Arm-/Y-/T-Maze	Longitudinal and automated learning and memory phenotyping of rodents living in a social group
Morris Water Maze	Spatial learning and memory testing without inducing forced-swimming/drowning stress
Open Field	Longitudinal, high-throughput, and automated monitoring of basal horizontal exploration, circadian activity, learning, memory, and cognitive function of rodents living in a social group
Conditioned Place Preference/Aversion	Positive reinforcement can be delivered as liquid reward
	Negative reinforcement delivered through air-puff instead of the classical electric foot-shock
Porsolt Forced Swim Test	Depressive- and anxiety-like behavioral phenotyping and drug-treatment effects without inducing forced-swimming/drowning stress
Vogel’s Conflict Test, Operant Conditioning (Fixed-/Progressive Ratio)	Door-nosepoke response replacing the classical lever response
	Negative reinforcement delivered through air-puff instead of the classical electric foot-shock
	Longitudinal, high-throughput, and automated monitoring in a social group
	Programmable fixed-/progressive-ratio schedule drinking

The IntelliCage system is a standard polycarbonate cage (55 cm width × 38 cm depth × 21 cm height) equipped with four triangular operant test chambers (15 × 15 × 21) fitted at each corner (Figure 1D). Animals are identified by the individual subcutaneously injected radio-frequency identification (RFID) tags (known as microtransponder; size: 12 mm × 2 mm) before being released into the IntelliCage system. Entry into each operant chamber is via the ring antenna which detects the animal’s unique RFID tags and records their visits. The round apertures on the walls of each chamber provide free access to water bottles. To date, mice have been reported to be supplied with tap water, sweetened water as a natural reward (i.e., sucrose or saccharin) (Radwanska and Kaczmarek, 2012; Iman et al., 2017; Heinla et al., 2018), aversive liquid (quinine solution) (Knapska et al., 2013; Smutek et al., 2014), or diluted liquid drug rewards (Radwanska and Kaczmarek, 2012; Marut et al., 2017; Skupio et al., 2017; Ajonijebu et al., 2018, 2019; Heinla et al., 2018). Small motorized doors at the aperture can be programmed too close to limit water access according to mice identification, time constraint, and conditioned action. Mice can be trained to perform a fixed or progressive ratio of nose-pokes at the door to allow access to water. The amount of liquid consumed is precisely measured by a lick-o-meter, while nose-pokes are measured by dedicated sensors. Three colored LEDs above the door in each corner provide visual cues. Aversive stimuli, or aversive reinforcement, employ the effective use of bitter tasting solution in one corner or brief air-puffs directed to the head of the mouse, therefore, eliminating the need for a more aggressive, painful, and fear-inducing stimulus (i.e., foot shock, vibration, loud noises). Four small triangular-shaped shelters are placed at the middle of the cage as a form of enrichment on which the mice could climb to reach for food (*ad libitum*). Shelters are red and transparent, but mice see red color as black so they are willing to hide inside which allows for their observation. The IntelliCage system also provides a continuous recording of the ambient variables (such as temperature and illumination) (Galsworthy et al., 2005; Lipp, 2005; Lipp et al., 2005).

### The IntelliCage System to Model Human Disorders

The ability and efficiency for longitudinal and high-throughput behavioral monitoring allow researchers to develop animal models of human disorders using the IntelliCage system. In the last decade, increasingly sophisticated and specialized IntelliCage protocols had been employed and validated to characterize mouse models for Huntington’s disease (HD), Alzheimer’s disease (AD), Down syndrome, SUD, autism spectrum disorder (ASD), and other neurological and neuropsychiatric disorders (summarized in Table 2).

**TABLE 2:** Summary of selected studies using the IntelliCage system to model human neurological disorders.

Disorder Model	Reference(s)	Strain and sex	IntelliCage parameters	Findings/description
Glutamatergic hyperfunction model	Kiryk et al. (2008)	GLT1-/-, GLT1+/- and WT ♀♂	Place preference	Demonstrated mild changes in the general activity and learning ability in mild GLT1 hyperfunction mice
			Place avoidance	Supplemented data for OF, EPM, and fear-conditioning tests
Huntington’s Disease (HD) model	Rudenko et al. (2009)	Tg-R6/2 and WT ♀	Free exploration Place and reversal learning Place avoidance Patrolling behavior Side alternation task	Validated IntelliCage for cognitive function study in HD mouse models
	Menalled et al. (2012)	zQ175-/-, zQ175+/- and WT ♀♂	Circadian pattern	Modified IntelliCage units with PhenocubeÔ
	Balci et al. (2013)	R6/2+/- BACHD+/- and WT ♀	Free exploration	Modified IntelliCage units with PhenocubeÔ
			Circadian pattern	Characterized disease profiles of 2 mutations
			Place and reversal learning	Validated IntelliCage for HD mouse models
Alzheimer’s Disease (AD) model	Codita et al. (2010)	Tg-ArcSwe ♀	Free exploration Place and reversal learning Novel object preference Novel smell (neophobia) Place avoidance	Demonstrated good test-retest reliability after approx. 10 months of standard housing Validated IntelliCage for longitudinal AD mouse models
	Sekiguchi et al. (2011)	Aβ-injected ddY ♂	Free exploration Nosepoke adaptation Place and reversal learning	Detected learning disturbance of Aβ-injected mice; reversed with yokukansen or donepezil treatment
				Validated IntelliCage for pharmacological studies of AD model
	Kiryk et al. (2011)	Tg APP.V717I ♀	Spatial memory Circadian activity Group learning	Demonstrated learning deficit in APP mutants and that the deficit was modulated by circadian activity and ameliorated by co-housed with WT mice
	Masuda et al. (2016)	AppNL/NL, AppNL-F/NL-F, AppNL-G-F/NL-G-F and WT ♀♂	Place and reversal learning	Validated Tg-App mice as AD model
			Place avoidance	IntelliCage data consistent with previously reported Tg-AD models
			Motor impulsivity	
			Delay discounting	
Down Syndrome model	Faizi et al. (2011)	Ts65Dn+/- and WT ♀	Place learning & avoidance	Detected avoidance learning deficit of Ts65Dn mice; reversed with β1-ADR agonist treatment
			Novelty exploration	Connected IntelliCage unit with novel satellite box
				IntelliCage data consistent with MWM and contextual fear-conditioning test
Substance Use Disorder (SUD) model	Radwanska and Kaczmarek (2012)	BALB/cJ and C57BL/6 ♂	Free exploration	Validated IntelliCage for addiction-related behavioral phenotyping and extended alcohol consumption (self-administration) in alcohol addiction mouse model
			Motivation for sucrose/alcohol	
			Impulsivity and anxiety test	
			Persistence for sucrose	
			Resistance to punishment	
			Alcohol self-administration	
			Withdrawal and relapse	
	Parkitna et al. (2013)	mGluR5KD-D1 and WT ♀	Alcohol self-administration	Adaptation of Radwanska and Kaczmarek (2012) alcohol abuse model with extended duration of alcohol access (>4 months)
			Abstinence	
			Circadian pattern	
	Marut et al. (2017), Skupio et al. (2017)	C57BL/6J ♀	Morphine self-administration CPP Motivation and persistence for morphine-seeking Resistance to punishment Withdrawal and relapse	Validated IntelliCage for morphine-induced behavioral phenotyping and morphine self-administration models Co-administration with glucocorticoid receptor antagonist attenuated morphine rewarding potential
	Iman et al. (2017)	Swiss albino mice ♂	Free exploration Sucrose preference and persistence Resistance to punishment Place & reversal learning	Mice were sensitized daily with morphine, THC, or mitragynine (psychoactive compound of *Mitragyna speciosa* or kratom leaves) Validated IntelliCage suitability as SUD mouse model for various widely abused substances
	Holgate et al. (2017)	C57BL/6 ♂	Alcohol self-administration Sucrose and alcohol preference	Social and environmental enrichment decreases ethanol preference and increases sucrose preference
	Ajonijebu et al. (2018, 2019)	C57BL/6 ♀	Cocaine self-administration Motivation and persistence for cocaine-seeking Withdrawal and relapse	Validated IntelliCage for cocaine-induced behavioral phenotyping and cocaine self-administration models
Traumatic Brain Injury (TBI) model	Muthuraju et al. (2013)	C57BL/6J ♂	Free exploration	TBI was induced with fluid percussion injury
				Locomotor assessments were compared in TBI-induced mice with/without normabaric hyperoxia treatment
	Vogel et al. (2020)	C57BL/6N ♀	Free exploration	TBI was induced with controlled cortical impact method
			Circadian pattern Place and reversal learning Place avoidance Hedonic/Anhedonic learning	IntelliCage data showed TBI-induced behavioral abnormalities and learning deficit consistent of post-TBI disorders (i.e., dementia, PTSD, and ADHD) with Barnes Maze
Autism Spectrum Disorder (ASD) model	Puścian et al. (2014)	C57BL/6 and BALB/c ♂	Free adaptation	Mice prenatally treated with valproic acid (VPA) to induce ASD
			Sucrose place and reversal learning	Detected significant reward-motivated learning deficit between VPA-treated C57BL/6 and BALB/c; valid for inter-species comparison
	Mitjans et al. (2017)	Ambra1^+/–^ and WT ♀♂	Pheromone-based social preference	Connected IntelliCage unit with two social boxes containing either fresh bedding or used bedding of mice from opposite gender (with pheromones)
Pneumococcal meningitis model	Too et al. (2014)	C57BL/6J ♀	Free exploration Nosepoke adaptation Light response test	Detected complex and dissimilar patterns of behavioral and cognitive changes in the Tg knockout mice
	Too et al. (2016a)	Tg-IDO1, Tg-IDO2, Tg-TDO and WT ♀	Patrolling behavior	
	Too et al. (2016b, 2019)	TLR2/4^–/–^ double deficiency and WT ♀	Place and reversal learning	
Coffin-Lowry Syndrome (CLS) model	Fischer et al. (2017)	Rsk2^*y/*–^ and Rsk2^*y/*+^ ♂	Free exploration Place and reversal learning Patrolling behavior Motor and cognitive impulsivity Vogel water lick paradigm (anxiety test)	Adapted Vogel water lick paradigm to IntelliCage using air-puff as punishment IntelliCage anxiety- and depression-like behaviors data consistent with standard behavior tests (EPM, LDB, OF, PST)
Neuropsychiatric disorder model	Heinla et al. (2018)	C57BL/6N and BALB/C ♀	Free exploration	Validated social mixing of two female mice strains in the IntelliCage environment for anxiety, stress, and neuropsychiatric disorder models
			Circadian pattern	
			Social competition and interaction	
			Saccharin preference (anhedonia test)	
Anesthetic drug interaction model	Zhu et al. (2008)	NM	Free exploration Spatial and reversal learning	14-days old mice were treated with isoflurane to induce memory deficit Demonstrated reversal learning deficit in isoflurane-treated mice
Hypoxia model	Lan et al. (2011)	C57BL/6 ♀♂	Free exploration Circadian pattern Place and reversal learning Cued punishment test	Hypoxia was induced by placing mice litters in an oxygen chamber IntelliCage data consistent with MWM test Validated IntelliCage for chronic, sublethal hypoxia model

*HD, Huntington’s disease; AD, Alzheimer’s disease; SUD, substance use disorder; TBI, traumatic brain injury; ASD, autism spectrum disorder; CLS, Coffin-Lowry Syndrome; NM, not mentioned; ♀, female; ♂, male; Tg, transgenic; WT, wild type; OF, open field; EPM, elevated plus maze; MWM, Morris water maze; PTSD, post-traumatic stress disorder; ADHD, attention deficit hyperactivity disorder; CPP, conditioned place preference; LDB, light/dark box; PST, Porsolt swim test.*

Furthermore, a modified and adjusted prototype of the IntelliCage system for rats has recently been tested with transgenic HD (Urbach et al., 2014) and valproate-induced autistic-like rats (Pelsöczi et al., 2020). The automated phenotyping using the IntelliCage and Phenomaster systems for rats successfully replicated the previously described behavioral phenotypes from conventional tests, and traced the novel physiological and behavioral aspects of transgenic HD rats, including circadian activity, anxiety, and rearing (Urbach et al., 2014). In addition, Pelsöczi et al. (2020) had successfully demonstrated the disrupted locomotion, circadian activity, and social hierarchy in a rat model of ASD, further indicating the IntelliCage system’s reliability and validity to measure rat ethological and activated behaviors. While extensive validations of the IntelliCage protocols for mice models have been reported thus far, its validation development seems slower for rat models. Rats have been described to commonly show more cautious locomotor exploration and avoidance features when placed in open field and maze-testing paradigms (Bertoglio and Carobrez, 2000; Alstott and Timberlake, 2009), thereby, could limit the interpretation using the IntelliCage data and warrants further extensive validation to merit a wider acceptance for research use.

## Previous Rodent Behavioral Studies With the Intellicage System

Substantial evidence has documented and recognized the practicality and effectiveness of the IntelliCage system for the short-term and/or long-term cognitive assessment of group-housed rodents. Earlier experiments with the IntelliCage system demonstrated its value for measuring spontaneous and simple conditioned behaviors. Ensuing studies developed and tested numerous parameters/protocols for the assessment of rodent social behaviors and cognitive functions, including spontaneous behavior and spatial navigation, learning and memory-related tasks, circadian activities, and place/drug preference or avoidance tasks. See Kiryk et al. (2020) for a more comprehensive description of the IntelliCage system parameters and protocols developed by approximately 80 research groups on a wide spectrum of rodent behaviors, to date.

### Spontaneous Behavior and Spatial Navigation

Spontaneous behavior, or free exploration, is considered as the mandatory first-stage assessment of rodent in the IntelliCage system; during which, all drink bottles are always freely accessible for approximately one week. The free exploration paradigm provides a unique opportunity to systematically assess novel and general environment exploration, and provides an initial screen for neophobia, habituation, gross motor deficit, coordination, and cognitive states in rodents. A dynamic representation of these states are indispensable for establishing individual baselines and detecting behavioral anomalies as the indicators of the animal’s general well-being, health, and emotional state in an unrestricted open-field environment (Bailey and Crawley, 2009; Fonio et al., 2009; Jirkof, 2014; Hohlbaum et al., 2018), as provided with the IntelliCage system. One of the early works by Galsworthy et al. (2005) explored simple exploratory behaviors and learning paradigms between two sympatric wild-caught rodent species (i.e., wood mice and bank voles). Parameters included were initial exploration during the first 90 min of introduction into a novel arena, total habituated activity levels throughout the subsequent 8 days (based on the number of corner visits and water consumption), and circadian patterns. This study acknowledged the IntelliCage system as a valuable behavioral testing module for both wild and in-laboratory rodents, as well as for inter-species comparison (Galsworthy et al., 2005).

The IntelliCage system is reportedly efficient for the long-term monitoring of female mice, while males may eventually require supplementary compartment barriers, housing about three males (Lipp et al., 2005). Small enrichment shelters were then added to the system design to limit any male aggressive or stressful behaviors that may confound the behavioral analysis. However, many ensuing studies use females for their phenotyping strategies in an attempt to avoid male aggression and dominance issues typical in social-grouped mice (Kiryk et al., 2020), thereby, creating a potential female bias and overlooking the potential sex differences (Weber et al., 2017). Thus, the underrepresentation of males in the IntelliCage system research must not be disregarded to improve scientific validity.

In another study, the IntelliCage system statistically revealed the indistinguishable differences in standardized inter-laboratory tests of exploration and activity parameters of F1 B6D2, C57BL/6, and DBA/2 mice, compared to the open-field, elevated Null-maze, water maze, and object exploration tests (Lipp et al., 2005). Safi et al. (2006) successfully adapted a simple Vogel water-lick paradigm in the IntelliCage system to assess anxiety and anxiolytic drug effects of the control and Diazepam-treated C57BL/6 female mice. The study reported an efficient and robust analysis of the individual behavioral parameters indicative of anxiety elicited by an aversive stimulus (i.e., number and duration of visits, licks, and nose-pokes following air-puff punished visit) (Safi et al., 2006).

Knapska et al. (2006) tested the system for place preference (by the acquisition of sweetened water at a specific corner) and avoidance (by avoiding a corner associated with air-puff) tasks to balance aversive versus appetitive conditioning effects within the central amygdala of C57BL/6 female mice. Further refinement of the balanced appetitive/aversive training has been provided by the study by Knapska et al. (2013), in which discrimination learning between sweetened water vs. bitter-tasting water provided in the two bottles in one corner was compared. The IntelliCage system has also been used to investigate mouse physiology and behavioral phenotypes in various mouse models as part of the spontaneous behavior and spatial navigation (Goulding et al., 2008; Jaholkowski et al., 2009; Mechan et al., 2009; Krackow et al., 2010).

### Cognitive Function

The most commonly used learning and memory-related protocol in the IntelliCage system is spatial/place learning for a specific IntelliCage corner associated with liquid reward. Thus far, results from the IntelliCage system, in the realm of cognitive and learning/memory functions, are parallel with those from standard behavioral assays, including Morris water maze and fear-conditioning tests (Kiryk et al., 2008; Konopka et al., 2010; Faizi et al., 2011; Vogel et al., 2020). A study by Onishchenko et al. (2007) focused on the long-term learning and memory effects of developmental exposure to methylmercury (MeHg) in pregnant C57BL/6 mice. The IntelliCage system was tested for spatial learning (learn to find water-rewarded corner) and reversal learning (learn to find newly placed water-rewarded corner) and patrolling behaviors. In the patrolling protocol, the water-reinforced corner was pre-programmed to change in a clockwise manner after each visit. Thus, mice had to learn to patrol to find the correct water-accessed corners, cued with a green LED light. This patrolling learning protocol in the IntelliCage system entails the involvement of mice visual discrimination, reference, and working memory challenge. This study also provided evidence that the IntelliCage system is more sensitive in the detection of behavioral alterations and learning paradigms in comparison to the Morris water maze, rotarod test, and forced swimming test (Onishchenko et al., 2007). The absence of mice social deprivation and any human interference with the IntelliCage system use may be the contributing factor to the sensitivity of the assessment.

More complex learning and memory protocols, including goal-directed behaviors (Gapp et al., 2014), serial reversal task (Endo et al., 2011; Kobayashi et al., 2013), chaining, and patrolling (Kobayashi et al., 2013), have also been successfully designed and employed using the IntelliCage system.

## The Intellicage System for Animal Model of SUD

Systematic phenotyping of rodent models in automated home-cage systems is presently receiving considerable attention as an effective means of monitoring general and complex activity parameters, as well as detecting perturbations in the neural circuitry function. These complex tasks are achieved while eliminating the tedious and error prone bias of human assessment over extended periods, allowing researchers to address and recognize larger arrays of behavioral outputs than those traditionally assayed. Indeed, the automated home-cage monitoring is a promising frontier for improving translational neurobehavioral research in rodents (Jhuang et al., 2010; Mingrone et al., 2020; Voikar and Gaburro, 2020), including in SUD models. In addition to the IntelliCage system, there are several other automated home-cage monitoring systems available at present (as summarized in Table 3).

**TABLE 3:** Summary of the available home-cage monitoring systems and their use in SUD in rodent models.

Home-Cage System	Detection System	Pros	Cons	Use in SUD models
ANY-Maze Behavioral Tracking Software (Stoelting Co.)	Video tracking	Video-tracking of animal activities in any types of cages and behavioral apparatus	Difficulty of tracking in low contrast environment, or in the presence of reflections	Guo et al. (2019)
		Longitudinal monitoring and high-throughput data	Track only one animal at a time (or two animals of different coloration)	Pushkin et al. (2019)
		Ease of use, simple set-up		
		Non-invasive procedures		
Activmetre (Bioseb)	Weight Platform	Platform can be used with standard rodent cages	Single housing	NA
		Detect slow/fast exploration, grid-climbing and immobility activity (e.g., grooming, nesting, rearing)	Limited data throughput	
		Longitudinal monitoring		
		Non-invasive procedures		
Phenotyper (Noldus)	Infrared Video tracking	Video-tracking of animal exploration, learning and memory, and cognitive function	Single housing Using thermal imaging camera Difficulty of tracking in low contrast environment, or in the presence of reflections	van Gurp et al. (2020)
		Built-in stimuli to detect wheel-running activity, avoidance/operant conditioning wall		
		Longitudinal monitoring		
		Non-invasive procedures		
IntelliCage (TSE Systems)	RFID Transponder	Social grouping for extended period	No video-tracking	Radwanska and Kaczmarek (2012), Parkitna et al. (2013), Marut et al. (2017), Skupio et al. (2017), Iman et al. (2017), Ajonijebu et al. (2019)
		Track multiple animals independently via RFID	No behavior recognition	
		High flexibility in programming tasks/schedules and parameters	Male aggression issue in social group	
		High-throughput behavioral data		
		Good replicability across labs		
		Add-on features such as running wheel and social chamber available		
Labmaster/Phenomaster (TSE Systems)	Infrared and Calorimetric tracking	Longitudinal monitoring	Single housing	Hay et al. (2019), McGonigle et al. (2016)
		High flexibility in programming tasks/schedules and parameters	Using thermal imaging camera	
		Track animal exploration, learning and memory, cognitive, and cardio-metabolic function	Difficulty of tracking in low contrast environment, or in the presence of reflections	
		Add-on features such as running wheel, operant conditioning wall, and climate chamber available		

*NA, not available.*

Thus far, a growing body of literature has attested the practical utilities and importance of the IntelliCage system in addiction-related mouse models, primarily in alcoholism research. Each IntelliCage corner chamber permits a voluntary oral consumption of liquid reward (via nose-poke), which is useful for self-administration paradigms, as well as the application of operant and Pavlovian conditioning tasks for studying the rewarding properties of various substances of abuse.

The IntelliCage system allows for mimicking different aspects of human behavior to meet the addiction criteria defined in the Diagnostic and Statistical Manual V (DSM-V) of the American Psychiatric Association (Hasin et al., 2013). The DSM-V recognizes that individuals are not all equally vulnerable to developing SUD, and that SUD is a pattern of symptoms. DSM-V sets a diagnostic threshold of 2 or more out of 11 criteria to be met. The IntelliCage allows for examining the following criteria: withdrawal, tolerance, craving, amount of consumption, and time spent on seeking. By analyzing these measures, it is possible to differentiate animals in terms of level of compulsive drinking into low and high drinkers (Radwanska and Kaczmarek, 2012; Stefaniuk et al., 2017; Beroun et al., 2018; Skóra et al., 2020). Radwanska and Kaczmarek (2012) designed the first longitudinal study of animal models of addiction using extensive IntelliCage system parameters in BALB/cJ and C57BL/6 male mice. The study successfully elucidated the behavioral traits associated with alcohol addiction, such as: (i) novelty-seeking (number of corner visits in the novel IntelliCage system); (ii) impulsivity (inability to withhold nose-pokes at rewarding corners); (iii) anxiety (suppression of reward consumption at air-puff associated corners); (iv) motivation and persistence for natural reward (i.e., 10% sucrose); (v) withdrawal; and (vi) relapse in mice for a span of 128 days. This study corroborated the IntelliCage system as a reliable tool for an efficient, high-throughput screening of mice addiction-prone behavioral traits. The data suggested that high levels of anxiety-related traits (i.e., low novelty-seeking, low resistance to punishment, increased compulsivity and impulsivity) predicted addiction-like alcohol drinking in mice (Radwanska and Kaczmarek, 2012). Parkitna et al. (2013) later adapted this alcohol abuse model for a 3-month assessment of ethanol self-administration, abstinence, circadian pattern of chronic ethanol consumption, and cue-induced alcohol relapse. Ensuing studies utilized the IntelliCage system paradigms to develop more complex learning and memory procedures in alcohol addiction models, including intermittent-access schedule (Smutek et al., 2014; Koskela et al., 2018), delay-discounting impulsivity (Szumiec and Parkitna, 2016), motivation for alcohol-seeking behaviors (Stefaniuk et al., 2017), alcohol-deprivation-induced effects (Thomsen et al., 2017), and cue-induced conditioning procedures (Koskela et al., 2018).

Additionally, the IntelliCage system has been used for oral morphine self-administration (0.1–0.5 mg/ml) in a progressive ratio nose-pokes, with the co-administration of dexamethasone [a selective glucocorticoid receptor (GR) agonist], and CPP paradigms to evaluate the GR effects on the rewarding properties of morphine in mice. This model represents a novel approach for investigating the behavioral and molecular mechanisms underlying opioid addiction (Marut et al., 2017). In a follow-up study, Skupio et al. (2017) evaluated mice compulsive morphine self-administration features, including progressive ratio nose-pokes, intermittent-access schedule, enhanced resistance to punishment, withdrawal, and reinstatement of morphine-seeking behaviors, for over 100 days. More recently, to induce and assess symptoms of compulsive cocaine intake, similar paradigms were adapted to the IntelliCage by Ajonijebu et al. (2018). Compared with the control animals, cocaine-addicted C57BL/6J female mice exhibited a higher preference for natural reward and failure to discriminate rewarded from non-rewarded corners, suggestive of significant learning deficits with a prolonged cocaine exposure (Ajonijebu et al., 2018, 2019).

Overall, considering the flexible task design and longitudinal monitoring in a social cage environment, the IntelliCage system indicates invaluable and promising abilities to be a novel model for short-term and long-term SUD studies for other substances of abuse. Therefore, based on this knowledge, our laboratory had successfully designed a new protocol (Iman et al., 2017), which was an adaptation from Radwanska and Kaczmarek’s mice alcohol addiction model (Radwanska and Kaczmarek, 2012), for the study of extended behavioral and cognitive effects of socially interacting Swiss albino mice chronically exposed to the widely abused substances, i.e., morphine, Δ-9-tetrahydrocannabinol (THC), and mitragynine, a major alkaloid of Thai medicinal plant, kratom or *Mitragyna speciosa* Korth leaves, with psychostimulant and opioid-like properties (Suwanlert, 1975; Ahmad and Aziz, 2012; Hassan et al., 2013; Saingam et al., 2013; Iman et al., 2017). In brief, data collected from our IntelliCage sensitization model (Iman et al., 2017) effectively presented the behavioral and cognitive impairment evoked by the chronic administration of morphine, THC, and mitragynine, which are consistent with the reports from previous studies using conventional animal addiction assays (Justinova et al., 2009; Lu et al., 2010; O’Brien et al., 2013; Harvey-Lewis et al., 2015; Yusoff et al., 2016; Vanderschuren et al., 2017; Hassan et al., 2019).

In addition, the water bottles and programmable conditioning corners in the IntelliCage system would allow researchers to study hedonic behavior and multitudes of spatial learning and memory function in drug-addicted rodents, as well as compulsive behaviors after being punished with an air-puff. Different colored LEDs feature can be utilized to study cue-induced drug memory. The system also differs from conventional tests of SUD in that it examines behavior over an extended period of time. Nevertheless, it is important to note that the main drawbacks of the IntelliCage system include its upfront setting-up costs, regular maintenance, absence of visual tracking of in-cage behavior and social interaction, as well as male aggression issue in socially grouped rodents. However, extracting together the current data and the analysis from previous addiction-related IntelliCage studies, we can assert that the IntelliCage system provides an effective and reliable platform to detect and characterize addiction-related behavioral phenotypes of rodent, chiefly mice, in a social dimension.

## Conclusion

In summary, current findings from the SUD mouse model characterize the IntelliCage system as a biologically valid, sensitive, and efficient system in the phenotypic detection of drug effects concurrently across multiple behavioral measures. Moreover, the IntelliCage system permits the assessments of behavior in a controlled environment for socially grouped rodents that minimizes human investigators’ interference. Concurrently, a fully valid model of substance addiction in the IntelliCage system can be further refined with more complex conditioning tasks and parameters, to complement other conventional behavioral assays. Thus, this platform can be beneficial in eliminating the bottleneck in rodent behavioral addiction studies. More importantly, it expands the opportunities to design better preclinical models of SUD to further elucidate the neurobiological mechanisms that contribute to addiction-related behaviors, as well as discovering related treatment options.

## Author Contributions

II, NY, and MMu conceptualized, drafted, and revised the manuscript. UT and NA contributed to the animal models of the SUD section. AN, JK, MMe, NJ, SM, MS, LK, and MMu critically reviewed the manuscript. All authors made substantial contribution to the review and approved the final manuscript.

## Conflict of Interest

The authors declare that the research was conducted in the absence of any commercial or financial relationships that could be construed as a potential conflict of interest.
